# Hormones as adaptive control systems in juvenile fish

**DOI:** 10.1242/bio.046144

**Published:** 2020-02-17

**Authors:** Jacqueline Weidner, Camilla Håkonsrud Jensen, Jarl Giske, Sigrunn Eliassen, Christian Jørgensen

**Affiliations:** University of Bergen, Department of Biological Sciences, Postboks 7803, N-5020 Bergen, Norway

**Keywords:** Hormone, Dynamic state-dependent model, Strategy, Growth, Survival, Allocation

## Abstract

Growth is an important theme in biology. Physiologists often relate growth rates to hormonal control of essential processes. Ecologists often study growth as a function of gradients or combinations of environmental factors. Fewer studies have investigated the combined effects of environmental and hormonal control on growth. Here, we present an evolutionary optimization model of fish growth that combines internal regulation of growth by hormone levels with the external influence of food availability and predation risk. The model finds a dynamic hormone profile that optimizes fish growth and survival up to 30 cm, and we use the probability of reaching this milestone as a proxy for fitness. The complex web of interrelated hormones and other signalling molecules is simplified to three functions represented by growth hormone, thyroid hormone and orexin. By studying a range from poor to rich environments, we find that the level of food availability in the environment results in different evolutionarily optimal strategies of hormone levels. With more food available, higher levels of hormones are optimal, resulting in higher food intake, standard metabolism and growth. By using this fitness-based approach we also find a consequence of evolutionary optimization of survival on optimal hormone use. Where foraging is risky, the thyroid hormone can be used strategically to increase metabolic potential and the chance of escaping from predators. By comparing model results to empirical observations, many mechanisms can be recognized, for instance a change in pace-of-life due to resource availability, and reduced emphasis on reserves in more stable environments.

This article has an associated First Person interview with the first author of the paper.

## Abstract (precis)

**Summary:** We combine physiological, environmental and evolutionary aspects of fish growth in a state-dependent model where the optimal regulation of growth and survival is achieved through hormonal regulation of behaviour.

## INTRODUCTION

It is a central aim of biology to understand how evolution has led to a specific organism design through natural selection. As Tinbergen (1963) pointed out, any trait can be understood both in terms of its mechanism and its evolution, and the philosopher Daniel Dennett (2017) has simplified this into two questions. If, for example, one is interested in fish growth, one may first ask ‘*How come* fish grow?’ The discipline of physiology has excelled at answering this type of question about underlying mechanisms, and has detailed triggers, pathways, intermediates, regulation, development and function from the molecular level to that of the organism. There is another set of explanations for fish growth if one asks: ‘*What* do fish grow *for*?’ ‘What for’ questions are about the adaptive significance, about the effects a trait has on survival, growth, reproduction and ultimately fitness. This evolutionary dimension introduces *purposiveness* to biology (Dennett, 2017): a goal-directedness that goes beyond blind chains of causation and transcends Hume's billiard balls that crash into each other. Rather, processes occur to fill a purpose, to obtain some kind of aim, for example feedback processes that restore homeostasis, or drives or urges that ensure survival, growth and reproduction. It must be emphasized that this is not an externally imposed or top-down purpose. It is a historic consequence of natural selection, where alleles with positive effects on survival and reproduction become more common in the gene pool, and their consequence is that organisms appear as goal-driven in their development, physiology, endocrinology, cognition and behaviour (Andersen et al., 2016; Budaev et al., 2019; Giske et al., 2013).

‘What for’ questions have been addressed by evolutionary ecology, life history theory and behavioural ecology, where empirical experiments and observations have often been inspired by theoretical considerations that have had one important limitation: they have typically ignored the proximate level of ‘how come’ questions. This was epitomized by Alan Grafen as the phenotypic gambit, inspired by the chess move where one makes a sacrifice to gain a longer-term advantage (Grafen, 1984). The phenotypic gambit was a methodological tactic where one tossed away all the mechanistic detail and simply assumed unbounded phenotypic flexibility. Then and now, this was in many cases a necessary assumption to be able to answer ‘what for’ questions. If models concluded that a trait had an adaptive advantage, the evolutionary ecologist would expect to see that trait to have evolved in real organisms in the wild. Any physiologist will immediately react to this as naïve and utterly unrealistic: real traits originate from genes, are built through biochemistry, obey the laws of physics, and any information used must emerge from a sensory organ or use local molecules directly. The organisms that live today share many design features that have evolved precisely because they allow flexibility within the boundaries set by these constraints (Giske et al., 2014). Over time this has led to descendant lineages that were more likely to evolve to fill new niches and respond to new selection pressures. The combination of ‘how’ and ‘what for’ questions, thus, reveals insights that one of them alone could not give (Sinervo and Svensson, 1998). On the other hand, the traditional separation of mechanisms from the individual's experienced selection pressures or ecological challenges tears them out of a natural framework of constraints. It also builds on the assumption that selection pressures influence underlying mechanisms much less than the actual behaviour or adaptation they produce (Garland et al., 2016).

In this paper, we focus on one architectural design feature for control of the organism – its hormone system – and with a model we ask several questions that we believe are useful to stimulate thought both among physiologists and evolutionary ecologists. For example, are key hormone systems sufficient to enact the adaptive flexibility seen in growth across different environments? Are there ways in which we can conclude that the major hormone systems are adaptive? If we treat the model as a thought experiment with unlimited flexibility in hormone expression, will observed correlations emerge between environments and hormones? Between hormones? And with ontogeny? The model is about growth and related survival in juvenile fish, but more importantly it aims to show how one can partly overcome the phenotypic gambit, not only in the model specification, but hopefully also by helping scientists from the two disciplines in asking and answering questions together.

It can be instructive to compare our process-based model with other modelling approaches to better see the type of questions we can reach for. One type of well-known modelling tool in physiology is the dynamic energy budget models [DEB, (Kooijman, 2010)]. These follow resources and energy in great physiological detail from ingestion to growth and reproduction, and may provide a good fit between predicted growth patterns and those observed in experiments and in the wild. Somewhat caricatured, one can describe DEB as ‘feed-forward bioenergetics’, where processes run as fast as resources or constraints allow. This perspective is similar to a combustion engine where the amount of gas fed into the carburettor determines the engine's power and speed. Models of feed-forward bioenergetics are designed to question what happens to metabolic processes if more or less food is processed, when external conditions change, for example temperature, or when there are extra costs due to e.g. disease or reproduction. These are analogous to how fast a car would go if it was loaded heavily with passengers, if cooling was difficult on a particularly warm day, or if one of the spark plugs didn't fire. In practice, DEB models can also lie in between and study how physiology changes to maintain a set growth rate (e.g. Lika et al., 2014), but DEB rarely questions the ultimate drivers that determine the adaptive growth rate under specific circumstances.

In contrast, our model optimizes survival through the juvenile phase, where the optimal growth rate emerges from the effects of growth on fitness. These may depend on the abundance of predators, food availability or duration of the growth season. Here, behaviour and physiology have to provide the resources required to achieve the target growth rate. This can be described as ‘by-demand bioenergetics’; a goal-driven control system that translates fitness incentives emerging in ecology into physiological responses that endow the phenotype with a performance to fulfil the set goal. This would be analogous to how hard the driver presses the gas pedal, which can depend on the speed limit or whether the driver is heading for the nearest hospital with a critically injured patient. The car is a tool to achieve a goal in the driver's mind, much like the physiology of an organism has potentials that can, if regulated appropriately, achieve fitness. It should be noted that DEB modelling acknowledges how organisms can have flexible phenotypes whereby energy use varies with environmental characteristics (see Lika et al., 2014 for an overview). While this is referred to as a ‘supply-demand’ spectrum (Lika et al., 2014), the focus is on how sensory organs and behaviour permit a flexible phenotype, not on how the resulting bioenergetics is purposeful or goal-driven because of its effects on fitness in the sense of Tinbergen (1963) or Dennett (2017).

There are several ways in which control mechanisms can regulate and interfere with the individual's bioenergetics. In a system that is goal-driven, a certain amount of energy will be directed to mechanisms needed to achieve the goal. The process of allocation of limited resources towards competing uses (Fisher, 1930) is therefore essential. Also, as resources must be acquired before they can be distributed, the acquisition rate is of importance. Often models deal with either acquisition or allocation. Here we combine the two-in-one model and under-one-control system. In this way, ‘by-demand bioenergetics’ can drive the phenotype towards its goal by increasing the goal-directed energy supply through acquisition and by reshuffling allocation among potential uses. Upregulating ‘by-demand bioenergetics’ in such a way can push the organisms into a state of fast growth and early maturation, or the opposite, as would best achieve fitness in a given environment. From an evolutionary point of view this would mean that life history changes from slow to fast or vice versa.

We have used the method of dynamic programming to connect short-term decision to fitness, as is common practice in evolutionary ecology (Clark and Mangel, 2000; Houston and McNamara, 1999). In the same tradition, we have also focused on a part of the full life cycle (juvenile growth up to 30 cm) by recognizing that survival during this phase is a prerequisite for achieving fitness later in life. To be specific about the goal-directness of growth in a proximate and mechanistic perspective, we treat the phenotype as having potential for a range of physiological rates, and focus on a simplified set of hormones as the control system. Because there are hundreds of hormones and associated signalling molecules in a typical fish or mammal, it was necessary to simplify to a level of complexity that is easier to grasp and analyse. We therefore first describe how we have interpreted the major regulatory routes that control growth in fish, and end up using three hormones and a neuropeptide that each play a specific role in our model. To a physiologist this simplification is most certainly incomplete as it definitely leaves out important elements, but our aim is to stimulate thinking, and we therefore ask the reader to follow us into this intermediate level of complexity. We now first describe how we have implemented our model, before we use the model to point to some interesting insights of the hormone system as adaptive, and ways forward to further bridging the proximate ‘how come’ and the ultimate ‘what for’ traditions in biology.

### Model

**Fig. 1.**
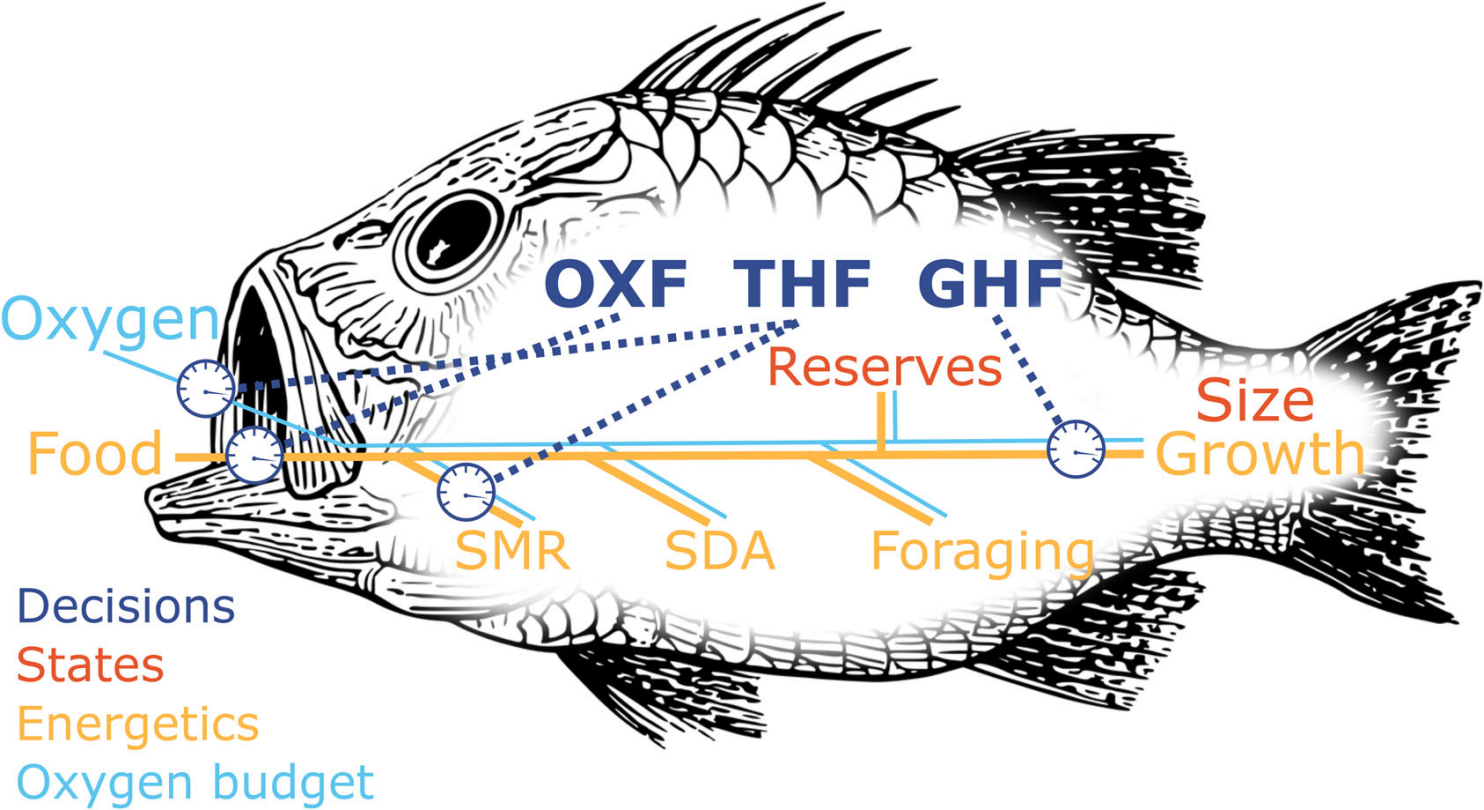
**Energetics and endocrinology of the model organism.** Energy from food is made accessible for the body by digestion (SDA). This energy is then used in metabolism to maintain life-supporting metabolic pathways (SMR) and supply the organism with oxygen. Also, activities like foraging require energy. The surplus is stored in reserves. Hormonal regulation determines the foraging intensity (OXF), increases or decreases of metabolism rates (oxygen uptake and SMR) and the allocation of resources to growth (GHF). Throughout the simulations, decisions regarding hormone levels are based on the two individual states of the fish – reserve size and body size.

The model organism is a generalized juvenile fish, and we choose parameters mostly from Atlantic cod (*Gadus morhua*), which is a well-studied species. The model follows juvenile fish as they grow through a size window where they typically remain immature. During this juvenile phase we let internal mechanisms like metabolism and growth be regulated by growth hormones (GHs), thyroid hormones (THs) and the neuropeptide orexin. They determine growth, metabolic rate and appetite, respectively, but importantly for the model they are also jointly involved in trade-offs related to risk (Fig. 1). The model also includes a role for the hormone leptin in signalling size of reserves.

We use a state-dependent dynamic model (Clark and Mangel, 2000). This algorithm first optimizes a strategy that can be considered the evolutionary adaptation to a certain environment. In the case of this model, the strategy is the optimal hormone levels for any combination of fish size and energy reserves. When the optimal strategy has been found, we investigate this adaptation by simulating individuals that live in the given environment and use the calculated optimal policy, and we record its trajectory of growth, hormone expression and individual states.

## RESULTS

**Fig. 2.**
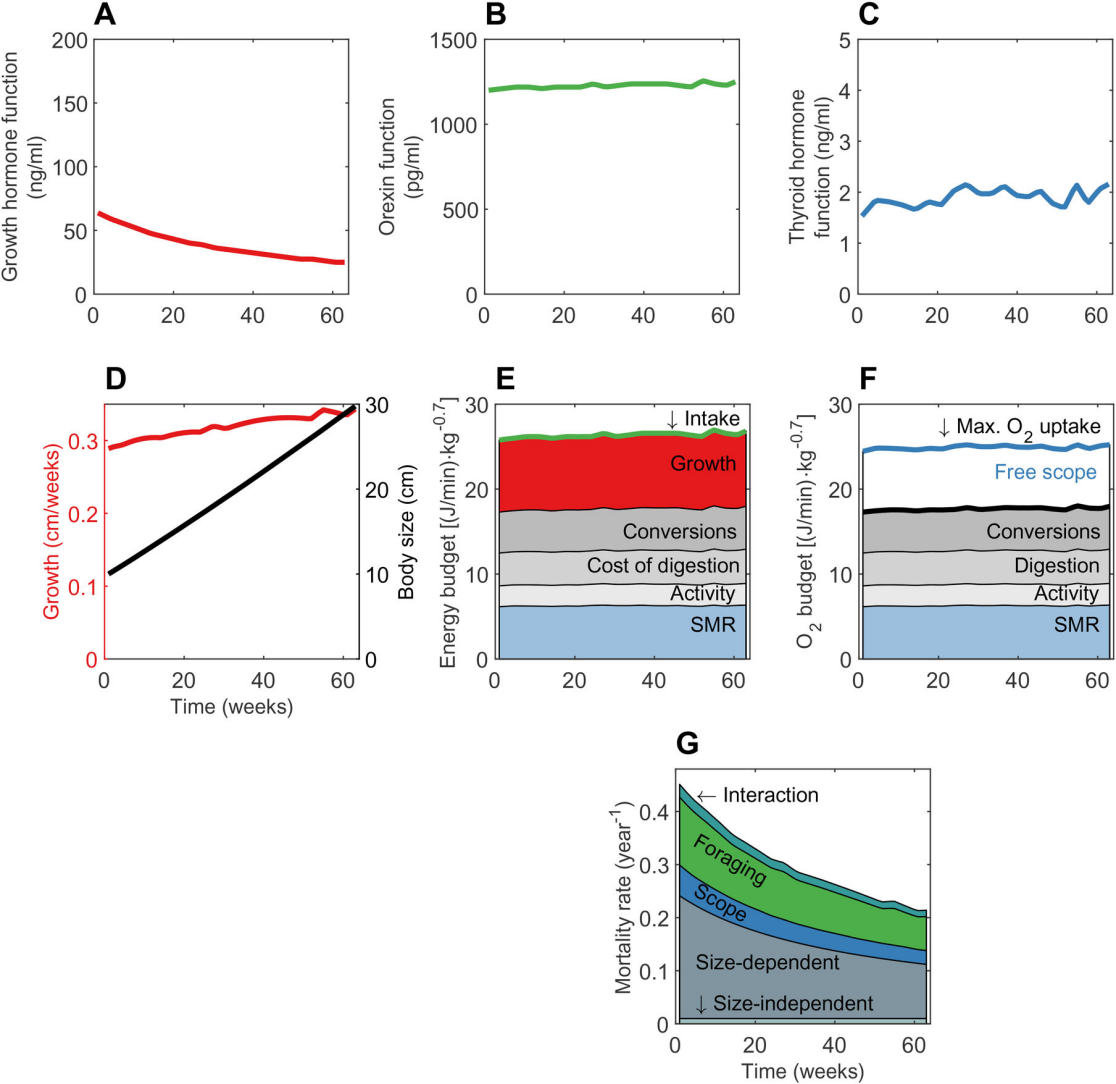
**Endocrine regulation, energy and oxygen budget, mortality and growth of juvenile fish in a stable food environment.** The simulation starts when the fish is 10 cm and ends at 30 cm, with the x-axis giving time (in weeks since 10 cm) in all panels. (A) Growth hormone function, (B) orexin function and (C) thyroid hormone function are given as a function of time. (D) Weekly growth and accumulated body mass, (E) energy budget, (F) oxygen budget and (G) mortality rate.

The optimal strategy for the hormone profile changed during the fish's growth phase, resulting in a near-linear length growth and decreased mortality rate over time (Fig. 2). While energy gain and oxygen budgets were relatively stable per unit of body mass, mortality decreased with size. The optimal level of growth hormone function (GHF) fell throughout the growth phase (Fig. 2A), but as the effect was relative to body size, the resulting growth in length was near linear (Fig. 2D).

The optimal level of orexin function (OXF; green) was relatively constant throughout the growth phase (Fig. 2B), which resulted in a stable food intake rate per unit of body mass. Energy from feeding was allocated to standard metabolic rate (SMR), specific dynamic action (SDA), soma, metabolic processes involved in conversion of food to reserves and growth, and the activity associated with searching for food (Fig. 2E). Since the food environment was not changing over time, the fish did not benefit from storing energy in reserves, but rather allocated all somatic investments towards structural growth (Fig. 2E).

There was some variation seen in the levels of thyroid hormone function (THF) over the growth period for the fish (Fig. 2C). This variation was too small to have a visible effect on SMR or maximum oxygen uptake per unit of metabolic mass (Fig. 2E,F). However, both SMR and maximum oxygen uptake for the individual increased due to increases in total body mass (data not shown).

The instantaneous mortality rate decreased during development (Fig. 2G), mainly because size-dependent mortality (grey area, Fig. 2G) is smaller for larger fish (Eqn 22). Foraging mortality (Eqn 23), scope-related (Eqn 24) and active-while-vulnerable mortality components (Eqn 25) also dropped. Foraging activity and free scope were relatively constant, hence changes in these mortality components were mainly due to lower predation risk with increasing size.

**Fig. 3.**
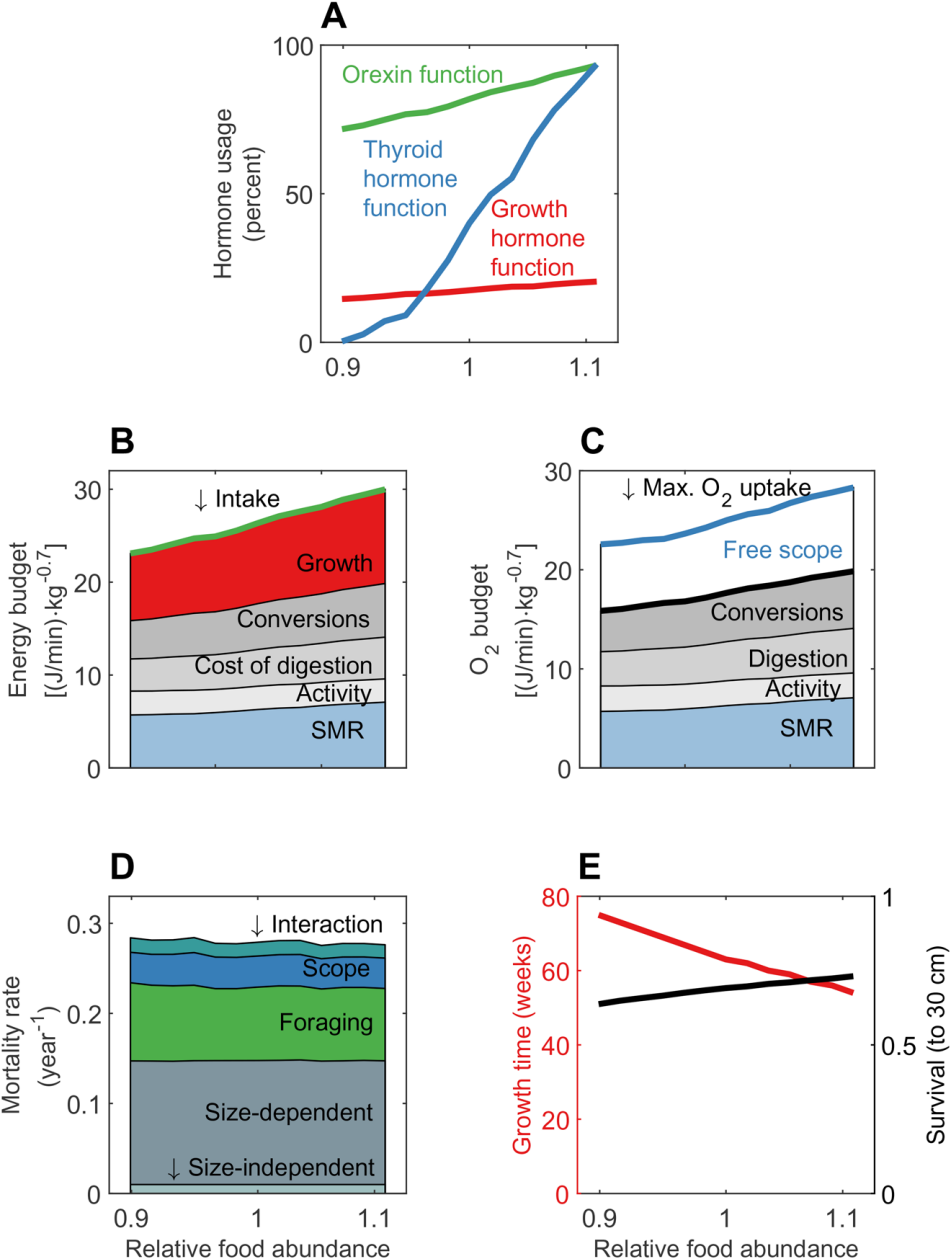
**Environmental influence on hormone levels, energy and oxygen budgets, survival and growth duration.** The x-axis is the same in all panels, with a gradual increase in food abundance relative to the average food environment as used in Fig. 2. Simulations of fish in 13 food environments were compared at individual length around 20 cm. (A) Hormone levels. (B) Energetic costs from growth and metabolism. (C) Free scope as the difference between maximum oxygen uptake and the sum of processes consuming oxygen. (D) Five different components contribute to mortality. (E) Growth time and survival over the entire juvenile life phase of the fish.

If we study how the optimal hormone strategies change along an environmental gradient in food availability, we see that the levels of OXF, GHF and in particular THF were higher in environments with more abundant food (Fig. 3A). Individuals in rich food environments grew faster, and had higher oxygen uptake and better survival probabilities. Faster juvenile growth requires increased energy intake, which resulted in higher SDA and conversion-related costs. Oxygen requirements also increased, which selected for higher THF levels that increased maximum oxygen uptake and secured free scope (Fig. 3C). THF also upregulated SMR, hence the optimal hormone level depended on the availability of energy in the environments and costs in terms of energy and mortality that came with gathering food. The energy allocation trade-off, between investments in maintenance and survival on the one hand and growth on the other, changed with food availability. Throughout the growth phase this trade-off was influenced by THF, which deducted energy to support a higher metabolic rate that in turn increased escape probability from predators. As energy was more accessible when food abundance was higher, activity costs were unchanged even when intake increased (Fig. 3B). Due to higher hormone levels, fish in habitats with high food availability had higher growth rates, intake and SMR (Fig. 3).

When comparing oxygen budgets (Fig. 3B), there was a slight increase in free scope from the poorest to the richest food environments. THF enabled the organism to increase its free scope despite higher oxygen use, thus permitting higher growth and foraging through the other hormones. Oxygen used for preparing metabolites for new soma reduced free scope, while THF worked against this process by elevating maximum oxygen uptake.

Simplified, GHF sets energetic needs, OXF meets the needs by determining foraging activity and providing metabolites for growth. The increased energy turnover has to be supported by THF, regulating maximum oxygen uptake to reduce mortality rate when energy is readily accessible and high turnover desirable (Fig. 3D).

Adaptations in hormone levels caused fish in rich environments to have a shorter juvenile phase (Fig. 3E). Despite similar instantaneous mortality rates (Fig. 3D), the probability of surviving to the end of the growth phase differed substantially between food environments because the duration of the growth phase was longer when food was scarcer.

## DISCUSSION

Most evolutionary optimization models of animal growth and survival focus on behaviour, size or other phenotypic traits while the internal regulatory processes are often ignored (Fawcett et al., 2014; Grafen, 1984). For fish, this includes social behaviour (Rountree and Sedberry, 2009; van der Post and Semmann, 2011), diel vertical migration (Burrows, 1994) and habitat choice (Fiksen et al., 1995; Kirby et al., 2000), but see Salzman et al. (2018). Here we take the opposite perspective, and study optimal internal regulation by hormone systems for animals that cannot choose their external environment. Obviously, most animals can do both at the same time, and habitat selection can have a direct impact on the physiological needs and priorities of the animal (Elton, 1927). But by removing the movement options in this model, we can isolate how internal mechanisms can be used to optimize trajectories of growth and mortality risk. We found variation in optimal hormone levels across different food environments and throughout ontogeny. The results in this paper are the outcome of an evolutionary optimization model based on an assumed connection between survival through a juvenile size window and lifetime fitness. We modelled adaptive evolution in three hormone functions, GHF sets the fitness-optimizing growth rate, OXF provides the required resources through appetite control and foraging, while the THF adjusts trade-offs between bioenergetics and survival. Effects of the hormonal control were evident in growth patterns, energy allocation, oxygen budget, activity levels and in survival.

Increased food availability enables organisms to grow faster, which is achieved by speeding up metabolism to accommodate increased physical and biochemical activity. Model fish adapted to high food availability by having higher optimal concentrations of GHF and THF than those adapted to food-restricted habitats (Fig. 3). Empirical studies testing for changes in hormone concentrations in relation to diet quantity focus on short-time experiments, often with feeding–starvation–refeeding cycles. Similarly to the predictions of the model, these generally find a positive correlation between hormone concentrations in plasma and the amount of food eaten by the fish (Lescroart et al., 1998; MacKenzie et al., 1998; Power et al., 2000; Toguyeni et al., 1996; Van der Geyten et al., 1998) or mammal (Herwig et al., 2008; Lartey et al., 2015; Nillni, 2010). Adaptive regulation of growth processes is indicated by the often-observed positive relation between ration size and growth rate in short-time experiments, e.g. in tilapia (Dong et al., 2015; Fox et al., 2010; Toguyeni et al., 1996), white sturgeon (*Acipenser transmontanus*) (Cui et al., 1996), gilthead sea bream (*Sparus aurata*) (Bermejo-Nogales et al., 2011), cod (Berg and Albert, 2003) and polar cod (*Boreogadus saida*) (Hop et al., 1997). Food availability is suggested to be one of the most important environmental factors influencing growth rates in fish (Dmitriew, 2011; Enberg et al., 2012; MacKenzie et al., 1998). We have not been able to find studies following hormone levels and growth rates of animals on differently sized rations throughout their growth phase.

Higher food availability in the model habitats resulted in higher optimal GHF levels and thus higher growth rates. Even if GHF in the model is a simplified version of the GH–IGF-1 axis, its response to stimuli like food availability resembles results from empirical studies. These studies show that concentrations of insulin-like growth factor-1 (IGF-1), a mediator of GH, decreased when food was less available (Bermejo-Nogales et al., 2011; Fox et al., 2010; Lescroart et al., 1998). Even though both GH and IGF-1 are essential for growth in natural individuals, growth rate typically exhibits positive correlations with IGF-1 but not with GH (see below). In addition to promoting growth in natural fish, GH has a lipolytic effect, amplifying the use of reserves during times of food restriction (Jönsson and Björnsson, 2002). In the model, we assumed stable environments and thus conflated the multiple effects of GH to a single effect on growth, thus, the lipolytic effect of GH cannot arise as a GHF-effect but would need to be prescribed through explicit assumptions. If the model were analysed in variable environments, we expect that it would be adaptive to build reserves, and if so, a lipolytic effect could emerge through a combination of GHF and OXF.

Increasing food availability in the environment triggered high growth rates via a combined effect of THF and GHF, although THF had no direct effect on growth in the model. Empirical studies account for the effect of hormones from both hormone axes on growth, which makes the emergent correlation in THF and GHF levels plausible. Somatic growth depends on several different processes, including bone and muscle growth, which in turn combine processes regulated by hormones such as T_3_ and IGF-1 from the two hormone functions. A study on tilapia documented a correlation between T_3_ and specific growth rates (Toguyeni et al., 1996). In mammals, T_3_ is involved in maintenance of chondrocytes and osteoblasts (Waung et al., 2012). It may have a direct effect on bone growth by local conversion and binding to thyroid receptors or an indirect effect via GH and IGF-1 (Nilsson et al., 2005). The interplay of TH and GH is also seen in chondrocyte development, in which a first phase is triggered by IGF-1 while the second phase depends on T_3_ (Robson et al., 2002). The GH dynamics follow the Dual Effector Theory, in which GH can act directly on cells or indirectly via IGF-1 (Jönsson and Björnsson, 2002). Despite their actions taking place at different locations in the bones or cells, or at different times during bone maturation, bones cannot grow if one of the hormones is missing. IGF-1 also plays an important role in muscle growth (Dai et al., 2015; Grossman et al., 1997), but to our knowledge effects of thyroid on muscle growth have not been documented.

Achieving high growth rate is always related to an increased demand for energy. This demand can be met by changes in energy acquisition and allocation, and in the model we saw that energy acquisition was higher in environments where food was more accessible (Fig. 3). Optimally, roughly a third of intake was allocated directly to growth while the remainder was lost to other metabolic costs on the way (Fig. 3B). The calculated average for six different teleost fish allocating metabolizable energy to growth at maximum rations of food was about 40% (Cui and Liu, 1990). Minimum and maximum allocation rates were 21.3% and 63.4%, respectively. Thus, the optimal allocation rate found in this model is within the observed range.

In many freshwater and marine systems, predation risk decreases with body mass (Barnes et al., 2010; Zaret, 1980), putting a high reward on fast growth for young individuals. From a life history perspective one would expect a decrease in length growth as the individual gets larger, due to fewer potential predators for larger fish (Byström et al., 2015; Persson et al., 1996) and how the increased survival prospects lead to slower optimal growth that put more weight on survival and the future. However, larger fish are more efficient feeders because they are less exposed to risk when they are foraging (Claireaux et al., 2018), countering the first effect. These two opposing forces explain the rather linear growth seen in the predicted juvenile growth from this model, an observation also seen in other adaptive models for the ontogeny of growth when acquisition is flexible (Claireaux et al., 2018; Jørgensen and Holt, 2013).

The challenges for the internal regulation mechanisms concerning storage of energy depend on the past, current and expected food environment. In natural environments, this can include preparing for environmental change by storing energy in reserves. In a stable food environment as in our model, building a reserve is not necessary and because it involves costs, it never becomes optimal and there will be no variation in condition factor among individuals. A modelling approach analysing energy allocation in environments varying in food availability (Fischer et al., 2011) concluded that energy storage can be advantageous, but depends on the size of current reserves and how variable the environment is. An empirical study of more than 40 fish species or genera found that fish in stable habitats often have lower condition factors than fish in more unstable habitats (Fonseca and Cabral, 2007). This supports the fact that fish from the completely stable model environment have minimal reserves.

As preparation for foraging, orexin A pathways are activated when food gets scarce, while in the model impacts of OXF on intake are strongest in rich environments. In the model, we saw a positive correlation between food availability and optimal OXF levels. Due to easily accessible energy in rich environments it was optimal to invest more into growth. This created a higher energy demand in the model fish, which was met by increasing OXF levels and foraging activity. From empirical studies, orexin A is known to affect the individual's energy budget on a short-time scale. It is negatively correlated to leptin, which serves as a proxy for the amount of stored energy in adipose tissue. Food restriction can result in higher orexin mRNA production, orexin receptor and neuron activity (Rodgers et al., 2002). This is also the case for ghrelin, acting together with orexin to prepare for and initiate foraging (Matsuda et al., 2011; Miura et al., 2007). Under fasting conditions, ghrelin levels can increase (Iwakura et al., 2015; Jönsson, 2013). Despite the trigger, low levels of stored energy, being the same in experiments and the model, the context in which the trigger occurs is different. This results in high levels of orexin A and OXF at different food abundances.

The shift described in our model cascaded from endocrinal changes affecting energy allocation and acquisition, oxygen budgets, growth and mortality risk, which in total caused a concerted response towards more rapid growth in rich food environments. Comparing poor to rich food environments, higher growth rates were supported by THF levels that upregulated SMR and increased maximum oxygen uptake. A positive correlation between metabolic rate and a range of traits contributing to rapid growth rate was found in Trinidadian guppies (*Poecilia reticulata*) (Auer et al., 2018), and this was also the case for our model fish.

Shorter growth periods with higher growth rates in rich food environments resulted in higher survival. Besides supporting growth, high GHF levels contributed to reducing size-dependent mortality by growing out of vulnerable size windows more quickly. High THF levels also lowered mortality by making escape once predators were encountered more likely to be successful. Thus, total mortality experienced through the growth phase was lower and survival at the end of the growth phase increased. To our knowledge, only GH excretion has been linked to mortality in empirical studies. The special interest assigned to GH is probably due to husbandry in which several land-living and aquatic animals have been genetically modified to excrete more GH and thus could grow faster to slaughtering size, e.g. coho salmon (*Oncorhynchus kisutch*) (Raven et al., 2008) and pig (Ju et al., 2015). Several studies have been conducted with both transgenic and hormone-implanted trout and coho salmon. Even if salmon fry can experience lower survival in the presence of predators (Sundström et al., 2005), several studies have found that fish treated with GH, thus having higher growth rates, have mortality rates similar to non-treated fish (Johnsson and Björnsson, 2001; Johnsson et al., 1999; Sundström and Devlin, 2011). In our model, these effects would come about because GH increased the demand for food, and the resulting increase in appetite and foraging involved risk-taking that elevated mortality rates.

The selection of fast-growing individuals over several generations may also influence endocrinology, as seen in salmon (Fleming et al., 2002). A better understanding of the combination of endocrinology and its consequences for growth is relevant also for animal breeding programs, including fish farming. Many physiological processes and traits are linked by the endocrinal network. Selecting one of those traits will inevitably lead to changes in the endocrinal network and affect other traits. For example, selection for high growth rates could increase oxygen use in metabolic processes to a level where fish cannot sustain other metabolic processes simultaneously, something that can be described as a limited ability to multitask physiologically. This means that the majority of available oxygen is used for metabolic processes supporting growth, while little or no oxygen is left to assure free scope as would be required for predator escape in the model. Other processes not modelled, like immune function, could suffer from constraints on oxygen uptake and use. A study on first-feeding salmon fry showed increases in mortality for GH-transgenic individuals under natural conditions (Sundström et al., 2004).

This model is a first step towards combining internal and external control of appetite with energy allocation, growth and survival in teleost fishes. To reflect mechanisms in nature, McNamara and Houston (2009) argue that models should consist of complex environments and simplified organisms. In our case, the environment is simple while the animal model is complex. Even with this simple one-factor environment, we saw a gradual change in optimal strategies for hormone expression that resulted in concerted trait differences between populations in poor and rich habitats. The model suggests an adaptive interplay of hormone functions, where GHF, OXF and THF act together to cause an adaptive life history strategy that balances growth and survival throughout the juvenile phase. Often, effects of the internal control by means of hormones have been studied in isolation from the selection pressure of the external environment. For the future, we suggest it is not sufficient to study only how hormones carry signals from tissues and sensory organs to control centres like the hypothalamus, nor only how the control centre influences the decision processes in the body at many levels. Rather, there is a need to view the entire organism as an evolved system, where key hormones mirror internal states and respond to external factors. Such decisions concern growth and survival, as in this study, but also other life history traits linked to maturation time or physiological preparations for maturation. It is this combination of emphasis on the endocrinal network in the model fish and its impacts on ultimate mechanisms such as growth and survival that is characteristic of the model. It makes the model a tool for understanding processes and mechanisms underlying adaptations of growth. We think this is a fruitful path where many studies may follow.

## MATERIALS AND METHODS

### Simplifying the hormone systems for model implementation

The central challenge for our model organism is to grow and survive up to adult size. Although a high number of hormonal molecules and mechanisms are used to dynamically control physiology and behaviour in natural fish, we single out three clusters: growth, energy acquisition and overall metabolism. We will refer to them as ‘functions’ to distinguish them from real molecules and complex pathways involved. When combined in a life history model, these functions also determine energy allocation to reserves. The main components of our mode are thus the GHF, the ORF and the THF. Leptin also plays a role as it contains information about the individual's energy reserves. Below we describe the main hormones involved in these axes and our rationale for simplification.

Decisions connected to growth influence the individual's life history. For example, fast growth enables organisms to reach sexual maturity relatively early in their lives and start reproducing before conspecifics. Growth processes can make up a major part of energy use. The main endocrinal driver of growth in fish and mammals is GH and its associated hormone cascade (Björnsson, 1997; Jönsson and Björnsson, 2002). Thus, in terms of ‘by-demand bioenergetics’, GH drives the fish towards sizes at which they can mature and reproduce, implying that fitness considerations have set up an energy-demand that the organism needs to fulfil.

Part of the growth processes initiated by the secretion of GH is the accretion of proteins and breakdown of lipids. Both processes influence the individual's condition, and they increase metabolism. To maintain its condition, the individual must increase its energy uptake through foraging. Appetite and the initiation of feeding behaviour are very complex processes, comprising the central nervous system and peripheral signals. An important group of neuropeptides are orexins, as they are produced in the hypothalamus where signals on condition and energy budget are integrated. Thus, orexins are the second step in the physiological response of the ‘by-demand bioenergetics’ model, as they regulate the individual's energy acquisition in order to fulfil the growth goal set by GH.

To achieve growth, GH as initiator and orexin as energy-suppliant are important factors influencing growth rate. Diving into growth mechanisms, there is another hormone and its associated cascade being ubiquitous for growth to happen: TH. Hormones from the GH cascade and the TH cascade make up a complicated network in which they promote each other's secretion, conversion, receptor activity and, in a chronological order, the developments of both cartilage and bone (Cabello and Wrutniak, 1989; Robson et al., 2002). Another reason for implementing a function on THs is their regulating effect on metabolism (see below). On the one hand, an upregulated metabolism may be an advantage when energy is abundant. This would push the individual into a state of high energy turnover. On the other hand, any increase in foraging exposes the individual to a trade-off between energy provisioning and foraging-related risk. The increased metabolism due to THs may weaken this trade-off by allowing for faster metabolism and higher potential activity level, in turn causing higher ability to escape in case of a predator attack. In terms of the ‘by-demand bioenergetics’ model, the individual's performance to fulfil the set growth goal is improved by higher energy turnover and oxygen uptake rates when conditions allow.

Starting with empirical data on stimuli, hormone regulation and effects, we now present the functions and mechanisms of these three clusters. Thereafter we will use this as background for the implementation in model code.

### The GHF

#### Effects

GH is expressed throughout life. In humans, maximal secretion is seen during puberty, then decreasing with age (Vermeulen, 2002; Zadik et al., 1985). GH seems to affect metabolism and body composition (Vélez et al., 2019; Vermeulen, 2002; Yang et al., 2018), but main effects are directed towards growth in bone (Nilsson et al., 2005; Robson et al., 2002) and muscles (Grossman et al., 1997). For fish, a relationship between GH levels and compensatory growth is suggested (Ali et al., 2003). To some extent GH also influences behaviour, either in a direct or indirect way (Jönsson and Björnsson, 2002). As growth rates can be constrained by environmental factors such as food availability, one would expect that GH levels and levels of its mediator IGF-1 fluctuate in line with seasonal variation. Any increase in GH-regulated growth processes depends on stimuli, e.g. information on the individual's current energetic status. In times of high food availability and increasing reserves, it is expected that individuals invest in growth as energy is relatively easily available. Food availability often varies predictably, for example algal blooms or increased vegetation in spring in temperate zones. Fluctuations, which might be stimulated by changes in photoperiod have been observed in reindeer (*Rangifer tarandus*) (Suttie et al., 1991, 1993) and Arctic char (*Salvelinus alpinus*) (Jørgensen and Johnsen, 2014).

#### Axis

GH production is controlled by a hormonal cascade, the somatotrophic axis. On top, GH-releasing factor (GRF) and/or somatostatin (SRIF) are released by the hypothalamus upon environmental or peripheral stimuli. These regulate the anterior pituitary activity, which alters the rate of GH secretion. GH effects are mediated by IGF-1 in most tissues. Both GH and IGF-1 can affect mechanisms in target tissues (Gatford et al., 1998; Peter and Marchant, 1995).

#### Stimuli

Through evolution the number of factors regulating GH release has decreased, while it is multifactorial in fish, regulation in mammals is mostly achieved by a ‘dual-control system’ (Gahete et al., 2009). The mammalian system consists of one main stimulator, growth hormone-releasing hormone (GHRH), and one main inhibitor, somatostatin (SRIF). Additional stimulators of minor importance are neuropeptide Y (NPY), ghrelin, exercise, and in some species leptin (Gahete et al., 2009; Hamrick and Ferrari, 2008; Kojima et al., 1999; Lanfranco et al., 2003). Leptin signals the current reserve size (Cammisotto and Bendayan, 2007), while ghrelin prepares the digestive tract for incoming food (Müller et al., 2015). In fish, a second main stimulator is pituitary adenylate cyclase-activating polypeptide (PACAP). Additional weaker stimuli come from thyrotropin-releasing hormone (TRH), gonadotropin-releasing hormone (GnRH) and others. Leptin does not exert a direct stimulus in fish (Gahete et al., 2009).

Melatonin (Suttie et al., 1992, 1991) regulates IGF-1 secretion. It is important to notice that one stimulus can have different effects on GH and IGF-1. This is for example the case in a study on fasted tilapia (*Oreochromis mossambicus*), where both body growth rates and body weight in males decreased due to fasting. IGF-1 levels correlated with growth rates, but GH levels were unchanged. A possible explanation is that available energy is used to cover basal metabolism first, while hormone levels are adapted to reduce or cease growth (Uchida et al., 2003). This is also the case for a diet experiment with Arctic char. Concentrations of GH did not reflect changes in body weight, but IGF-1 concentrations did (Cameron et al., 2007). Unchanged or even elevated levels of GH can be part of a fasting response in which GH impels lipolysis and prevents protein degradation (Richmond et al., 2010).

Inhibition of GH is also exerted via IGF-1 in a long feedback loop in both fish and mammals (Gahete et al., 2009).

### The OXF

#### Effects

Orexin is a neuropeptide known from humans (Kalamatianos et al., 2014; Oka et al., 2004; Tomasik et al., 2004), pigs (Kaminski et al., 2013), rats (Dube et al., 1999) and fish (Facciolo et al., 2010). There are two types of orexin, A and B, which have several effects, including feeding-related and behavioural effects (Cai et al., 2002; Rodgers et al., 2002). Orexin A stimulates foraging in goldfish (*Carassius auratus*) (Volkoff et al., 1999) and rats (Dube et al., 1999; Rodgers et al., 2000). Positive correlations between caloric demand and both orexin A and B exist for children (Tomasik et al., 2004). Observations of mice injected with orexin A and B revealed no effect of orexin B on food intake, while orexin A increased food intake and metabolism (Lubkin and Stricker-Krongrad, 1998). One mechanism by which orexin can act on food intake is via regions in the brain such as the arcuate nucleus (ARC) (Rodgers et al., 2002), where also leptin influences energetic processes in the body. It has also been suggested that foraging activity is increased by delaying satiety, as shown for low-dose treatments in rats (Rodgers et al., 2000). Effects not related to feeding include a general arousal, reduced pain perception, increased locomotion, etc. (Rodgers et al., 2002), and many of these can be seen as enabling for foraging. Despite both orexins being present in a variety of organisms, the effect of orexin A on feeding behaviour seems to be much stronger than that of orexin B (Edwards et al., 1999; Haynes et al., 1999; Nakamachi et al., 2006; Sakurai et al., 1998). Beside the direct influence of orexin neurons by leptin via leptin receptors (Funahashi et al., 2000; Sakurai, 1999), orexin neurons are also influenced by neurons responsive to NPY and Agouti-related peptide (AgRP) (Sakurai, 1999). NPY/AgRP-neurons are of first-order, thus they collect incoming information on the individual's energetic status and translate this information into a downstream response (Barsh and Schwartz, 2002; Loh et al., 2015).

#### Stimuli

Factors influencing the secretion of orexin describe the body's current state in terms of energy availability. A stimulating factor reported for rats is the fall in plasma glucose levels, eventually in combination with an empty stomach (Cai et al., 2002, 1999). However, a study on rats with insulin-induced fall in plasma glucose only showed an increase in hypothalamic orexin B (Cai et al., 2001). When energy is available to the organism, orexin secretion is inhibited. A signal of ingested food can be gastric distension (Cai et al., 1999). Leptin receptors have been found linked to orexin neurons in rodents and primates (Horvath et al., 1999) and may decrease the secretion of orexin in the hypothalamus (Kalra et al., 1999). Orexin A is believed to be part of a short-term response to ensure energy balance in the body (Cai et al., 1999; Rodgers et al., 2002).

Orexin effects in fish are similar to those in mammals (Matsuda et al., 2012) and they have been detected in several fish species (Miura et al., 2007; Nakamachi et al., 2006; Volkoff et al., 2003). Most experiments are done on goldfish (Penney and Volkoff, 2014), but also cavefish (*Astyanax fasciatus mexicanus*) show an increase of orexin A in relation to food intake (Penney and Volkoff, 2014). An interplay between orexin and ghrelin is suggested for foraging initialisation, in which ghrelin stimulates food intake and mediates orexin effects (Miura et al., 2007; Penney and Volkoff, 2014). Ghrelin is known from several fish species (Matsuda et al., 2011). In mammals, an increase in ghrelin-concentrations can be observed before food intake (Müller et al., 2015). In fish, it seems that patterns in ghrelin secretion are more species-specific. Several species show increases, as in mammals, but also decreasing concentrations are found (Jönsson, 2013; Penney and Volkoff, 2014; Rønnestad et al., 2017). Despite differing mechanisms, it seems that the positive effect of ghrelin on foraging is similar across fish species. In addition to direct effects on feeding, orexin also exerts effects on other behaviours, like waking and locomotion (Rodgers et al., 2002). Its comprehensive effects are supported by the widespread presence of orexin receptors in the brain (Sakurai, 1999).

### The THF

#### Effects

In mammals and fish, THs are major factors regulating metabolism and development. The hormones affect brain development (Di Liegro, 2008), metamorphosis (Youson et al., 1994) and, in combination with GH, bone growth (Nilsson et al., 2005; Robson et al., 2002). Throughout life the basal metabolic rate is regulated by TH (Heilbronn et al., 2006; Herwig et al., 2008; Kitano et al., 2010; Webb, 2004). Due to their effect on metabolism they also play an important role in preparing organisms for seasons of low temperature and food availability [e.g. in red deer (*Cervus elaphus*) (Kuba et al., 2015), red knot (*Calidris canutus canutus*) (Jenni-Eiermann et al., 2002), reindeer (Bubenik et al., 1998) and white grouper (*Epinephelus aeneus*) (Abbas et al., 2012)]. Consequently, some seasonal variation in circulating hormone levels can be detected. A reduction of up to 30% in basal metabolic rate in the absence of TH is documented for endotherms, and this reduction can be linked to thermogenesis (Heilbronn et al., 2006; Mullur et al., 2014; Silva, 2003). Non-thermogenic effects include the regulation of body weight and metabolism of triglycerides and carbohydrates (Mullur et al., 2014; Varghese and Oommen, 1999; Varghese et al., 2001). In both mammals and fish, an impact on cardiac output is documented (Carr and Kranias, 2002; Little and Seebacher, 2014), and effects of TH on resting hearts have been shown in zebrafish (*Danio rerio*) (Little and Seebacher, 2014). As cardiac output contributes to maintain aerobic scope, TH also impacts the animal's ability to sustain sufficient oxygen uptake under changing temperatures (Little and Seebacher, 2014).

#### Axis

TH secretion depends on a hormone cascade sustaining relatively constant circulating hormone levels. On environmental or peripheral stimulation, TRH is secreted by neurons in the hypothalamus. In mammals, it promotes release of thyroid-stimulating hormone (TSH) from the pituitary. In fish, the relation between TRH and TSH is not as clearly defined (Abbott and Volkoff, 2011; Chatterjee et al., 2001). In both mammals and fish, TSH acts on the thyroid gland, the actual place of TH production, which is stimulated to release TH into the blood. Those are mainly thyroxine (T_4_) but also triiodothyronine (T_3_), which differ in the number of their iodide ions (Han et al., 2004; Zoeller et al., 2007). Relatively constant hormone levels in the body are accomplished by negative feedbacks in the hormone cascade (Fekete and Lechan, 2014; Zoeller et al., 2007). TH are mainly eliminated from the blood by deiodination in the liver (Malik and Hodgson, 2002; Zoeller et al., 2007). The first deiodination-process forms the bioactive T_3_ from T_4_. There is also some evidence on the direct effect of TRH on feeding and locomotor activity (Abbott and Volkoff, 2011).

Target tissues, such as the brain, bones and kidneys, contain different kinds of metabolic enzymes, deiodinases, to remove iodide from the hormones (Friesema et al., 1999; Miura et al., 2002). Biologically inactive T_4_ has to be converted to T_3_ in order to have an effect on tissues (Zoeller et al., 2007). There are three deiodinases, which successively can remove iodide ions to form T_3_, T_2_ and T_1_. An inactive form called reverseT_3_ can also be produced (Zoeller et al., 2007). Although it seems that most studies are concerned with the actions of T_3_, there is some evidence on effects of T_2_ (Lanni et al., 2001) and T_4_ (Robson et al., 2002).

#### Stimuli

Several factors stimulating the release of TH have been identified, e.g. leptin (Abel et al., 2001; Herwig et al., 2008; Nillni et al., 2000) and insulin (Lartey et al., 2015). Leptin transfers information based on individual fat stores to the brain (Cammisotto and Bendayan, 2007) where the signal influences secretion of TRH positively (Fekete and Lechan, 2014). Inhibiting effects are known from stress (Silberman et al., 2002), exhaustive exercise (Hackney and Dobridge, 2009) and melatonin (Ikegami and Yoshimura, 2013; Ono et al., 2008).

### Simplification of hormones functions in the model

#### GHF

As our interest is in hormone strategies for growth, the GH cascade is reduced to one variable in the model. This is a proxy for a fish's IGF-1 blood plasma concentration and regulates the amount of energy drained from reserves and used for building all kinds of somatic structures, including bones. The complex hormonal network of ghrelin, leptin and the somatotrophic axis is resembled in the interaction of GH and current body states, notably energy reserves and satiety. In the model the axis, its effects and stimuli are referred to as the GHF (Eales, 1988).

#### OXF

The OXF represents stimuli, hormone secretion and effects of orexin as one value. For the model, only orexin A is regarded. To simplify its effects, the OXF only affects foraging behaviour in a positive manner. Foraging is assumed to include a series of other effects, such as arousal and increased locomotion, and in the model these are reflected in energetic foraging costs. Motivated from behavioural ecology, there comes a mortality cost with increasing foraging activity as looking for food involves potential encounters with predators. In the model we consider the longer-term effect of the ORF as a proxy for the mean orexin A concentration in the body during this period of time. Orexin A concentrations vary on a timescale much shorter than the time resolution chosen here. For example, concentrations can vary with a daily cycle (Grady et al., 2006), and furthermore during the oestrous cycle (Kaminski et al., 2013), body weight reduction (Bronsky et al., 2007) and prepro-orexin mRNA increases in fasting individuals (Cai et al., 1999). Here we concentrate on the mean effect orexin A has on stimulating foraging activity over the course of one model time step.

#### THF

For the purpose of the model, a long-term effect of TH is of interest. Stress from predation, insulin and other factors that signal environmental or individual conditions on a short timescale are hence neglected. In the model the thyroid cascade is reduced to a simple factor resembling blood concentrations of bioactive T_3_. Negative feedback and elimination in order to receive relatively constant concentrations of TH in the body are disregarded; this is also done for the minor effect of T_2_ and T_4_. Effects of TH are reduced to an influence of thyroid on metabolism. Metabolism is regarded as the mean turnover of energy from food to reserves, soma or activities. The influence of TH on metabolic mechanisms in the model is summarized in a positive linear correlation between TH concentration and SMR. While this correlation is regarded as the cost of TH, a benefit comes with the positive linear correlation between TH and potential oxygen uptake, for example partly mediated through heart function. An increase in potential oxygen uptake (caused by TH) results in a greater free aerobic scope, which in turn contributes to better swimming ability and higher escape probability in case of a predator attack. Non-metabolic processes such as brain development or metamorphosis are not part of the model. As the ‘thyroid axis’ in the model covers response to stimuli, the hormones themselves and their effects, it is called THF (Eales, 1988).

Leptin is not modelled directly, but signals size of the energy reserve and therefore allows the model to have a reserve as an individual state that can potentially influence the levels of the other hormone functions.

### Model description

Hormones regulate physiological and behavioural processes, and these in turn achieve benefits and incur costs that may depend on the environmental conditions and the state of the organism. When we say we model hormones, it is therefore the effects of hormones that are in focus, in our case their consequences for growth and survival of juvenile fish. We first give the four central equations that describe growth and survival in our model, then detail the underlying processes. Throughout, capital letters are used for array variables that describe the organism and may change over time or with state (listed in Table S2), while lowercase is used for parameters that have a specific value (listed in Table S1). Greek letters denote the strategies, i.e. the hormone levels that the model optimizes. Central aspects of energetics, oxygen use and hormonal regulation are visualized in Fig. 1.

The model characterizes fish body mass *W* [g] as being separated into two components, where the structural body mass *W*_structure_ [g] grows irreversibly. On top of that are the energy reserves *R* [J] that can be built or tapped, having an energy density *d*_reserves_ [J g^−1^]:(1)
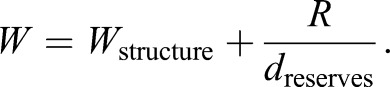
The distinction between irreversible structure and dynamic reserves is common for many models, including DEB (Kooijman, 2010). Growth Δ*W*_structure_ [g week^−1^], the irreversible increase in structural body mass, depends on the level *γ* [ng ml^−1^] of the GHF relative to its maximum value *γ*_max_ [ng ml^−1^], current structural weight and *k*_growth_ [week^−1^], which sets the upper limit for proportional increase in structural body mass per time step (weeks):(2)
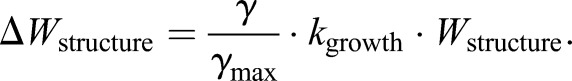
From the bioenergetics budget it follows that all energy taken up as food *I* [J min^−1^] is used for either metabolic processes *P* [J min^−1^] or to pay energetic costs of building tissues *C* [J min^−1^]. These new tissues include both new soma and changes in reserves:(3)
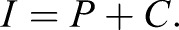
The details of *I*, *P* and *C* are described in detail further down. Hormonally, *I* is controlled by the OXF, *C* by the GHF through tissue costs of growth and *P* is influenced by the extra metabolic costs of expressing the THF.

The last central equation relates to survival probability *S* [week^−1^], which is given by *S*=e^−*M*/52^ where *M* [year^−1^] is the total mortality rate compounded by several components:(4)
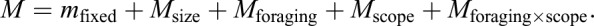
Here *m*_fixed_ is a constant irrespective of size, state or strategy. *M*_size_ is a predation rate that declines with size. *M*_foraging_ is predation resulting from exposure while foraging. *M*_scope_ is increased vulnerability when the individual's overall metabolic rate is close to its maximum aerobic capacity, because it is then harder to escape an attack. Similarly, *M*_foraging×scope_ is extra mortality when the individual exposes itself to predators while it is exhausted, which would put it in double jeopardy. The THF affects both *M*_scope_ and *M*_foraging×scope_.

Understanding the model requires that the equations above are interpreted in light of three key trade-offs that we describe here and give details and equations for further down.

First, the energy requirement of growth and everything else has to be met by foraging for food, which involves taking some level of extra risk (Krause and Godin, 1996; Lima and Dill, 1990; Sih, 1992). A resting fish often seeks safety in a shelter but needs to leave this to seek habitats where prey, and most often predators, are more common. Acquisition of more food thus involves more encounters with predators, and when food is scarce the fish needs to search for longer and expose itself more to forage the same amount.

Second, aquatic breathing is rapidly limited by surface-to-volume ratios and gas diffusion, even for small organisms. Although respiratory organs such as gills have evolved to overcome these constraints, there are physical limits to permissible total metabolic rate (Priede, 1985). Maximum aerobic capacity is often measured on fish that swim in respirometers, but digestion and growth are also variable processes that contribute to total metabolic rate. When the overall level of metabolic processes requires a lot of oxygen, the fish is quickly exhausted and therefore less efficient at evading predators should it encounter one.

Third, a trade-off that has received less attention is how spending energy can help an organism to manage, mitigate or reduce risk. It is known that immune systems incur energetic costs, and that the optimal level of immune function depends on energetic status, the risk of infections and availability of resources. Here we use thyroid regulation of metabolic level to achieve a similar exchange between energy and risk. The model assumes metabolic level can be upregulated by thyroid at an energetic cost (subject to trade-off 1), and the extra metabolic capacity is modelled as an elevated aerobic scope (alleviating trade-off 2). Consequently, the model allows metabolic rate to vary systematically between ecological settings.

We use a state-dependent model to find the optimal hormonal control of acquisition and allocation of energy. This type of mechanistic model finds the evolutionary endpoint (beyond which further changes cannot improve fitness) for a given environment. The model first uses dynamic programming (Clark and Mangel, 2000; Houston and McNamara, 1999) to find the optimal hormone expression for each combination of the individual's states. This is referred to as the strategy, as it contains information about what the individual optimally should do under each circumstance and in each state. The individual states included are the body length of the fish and its energy reserves. Thereafter, an individual that makes use of the optimal strategy according to its current individual state is simulated. We record its trajectory of growth, physiology, behaviour and risk-taking to quantify and analyse effects. The model optimizes the state-dependent trajectory of the three hormones (GHF, OXF and THF) by maximizing juvenile survival between 10 cm and 30 cm body length. The time steps are set to one week to represent typical dynamics of hormone levels and growth processes, which means that more rapid processes like behaviours are not modelled in minute-to-minute detail but for their cumulative effects at a weekly scale. The model describes growth of a juvenile fish in environments with constant food availability, and we compare several different environments in our analyses.

### Energy budgets and metabolic rate

The total metabolic rate *P* [J min^−1^] is the sum of all respiratory processes, all with unit joules:(5)
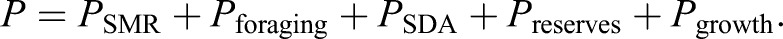
Here *P*_SMR_ [J min^−1^] is the standard metabolic rate, *P*_foraging_ [J min^−1^] the swimming cost of foraging behaviour, *P*_SDA_ [J min^−1^] the cost of digestion and energy uptake (SDA) until the resources are available in the bloodstream, and *P*_reserves_ [J min^−1^] and *P*_growth_ [J min^−1^] the metabolic costs of converting between resources in the bloodstream and reserve and structural tissue, respectively.

On top of that, the organism uses its digested resources for incorporation as new structural tissue (*C*_growth_ [J]) or by adding to or using from energy reserves (Δ*R* [J]). The net rate *C* [J min^−1^] of such incorporation of energy into tissue is thus:(6)
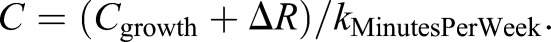
Note that while *P* and *C* both contribute to the individual's energy budget (Eqn 3), only *P* uses oxygen through aerobic respiration (Eqn 24).

SMR scales allometrically with body mass as the fish grow from juvenile to adult size. Other contributors to an individual's overall metabolic rate are factors like locomotion, digestion and growth, and many of these may change with ontogeny (Mozsár et al., 2015).

The model uses variants of SMR in several ways. What is measured experimentally as SMR and that we refer to as *P*_SMR_ is the standard oxygen consumption of the organism's total body mass as it is affected by the level of the THF. We first consider the baseline level of SMR at a mean level of THF expression as:(7)
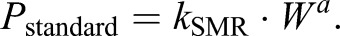
Here, *k*_SMR_ has unit [J min^−1^ g^−*a*^]. *P*_standard_ can be up or downregulated under the influence of THF, modelled as the concentration *τ* [ng ml^−1^] and relatively to a maximum concentration *τ*_max_ [ng ml^−1^]:(8)
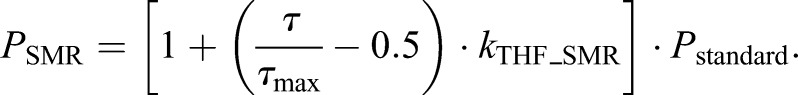
Here, *k*_THF­_SMR_ determines the strength of the effect of THF on metabolic rate, or in other words, the energetic cost of upregulating the scope for metabolic activity. It is *P*_SMR_ that enters the individual's metabolic rate (Eqn 5).

When we model food intake as a multiple of SMR, it is unlikely that a chubby individual has higher foraging success per time and energy investment compared to a leaner fish, so we scale food intake with *P*_structure_, a measure of SMR calculated from the lean body mass only and not affected by THF:(9)
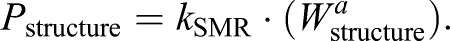


### Foraging and digestion

Energy from foraging is ultimately used to drive all energy-dependent processes in the organism. We model foraging as controlled by appetite through the OXF where the relative concentration of OXF (*α*/*α*_max_) is proportional to the target intake rate *I* of the individual, which is expressed as:(10)
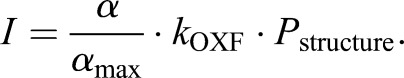
Intake *I* [J min^−1^] is defined as metabolizable energy absorbed by the gut; urinary and fecal loss of energy are implicitly included in the dimensionless coefficient *k*_OXF_ (Bureau et al., 2003). Here *P*_structure_ is a standardized metabolic rate of the lean body mass, explained in Eqn 9 above, used because it is unrealistic that having large reserves contributes to more efficient foraging.

The foraging behaviour *B*_forgaging_ [dimensionless, given in multiples of *P*_structure_] required to meet the energetic demand depends on food availability in the environment. We first rescale foraging intake to multiples of SMR, which allows us to find the level of foraging behaviour needed to meet the orexin-regulated appetite in a certain food environment *E* [dimensionless]. We assume that food is quicker and safer to find in rich food environments:(11)
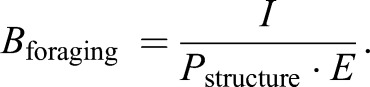
The cost of foraging activity (*P*_foraging_) is proportional to foraging activity and SMR with a coefficient *k*_foraging_ [dimensionless]. Physical activity during foraging requires moving the whole body, including soma and reserves, so SMR is based on total weight.(12)
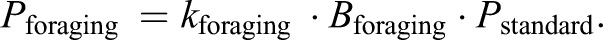
Food eaten is processed by the digestive system and taken up into the bloodstream. Specific dynamic action SDA (*P*_SDA_), representing the cost of digestion, is the product of intake and a constant *k*_SDA_ [dimensionless].(13)
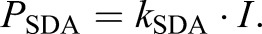


### Growth and reserves

Structural weight (*W*_structure_) is calculated based on length *L* [cm] using Fulton's condition factor for lean fish (*k*_Fultons_min_, [0.01 g cm^−3^]):(14)
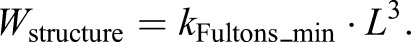
Likewise, maximum storage depends on body size and is calculated from the difference between maximum (*k*_Fultons_max_, [0.01 g cm^−3^]) and lean condition factor, and the energy density of the reserves (*d*_reserves_, [J g^−1^]):(15)
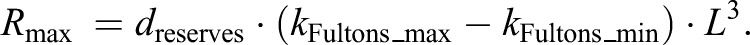
The cost of structural growth *C*_growth_ follows directly from the amount of new tissue produced (Eqn 2) and the somatic energy density *d*_structure_ [J g^−1^]:(16)
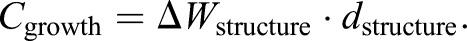
While reserves may vary in size, the model assumes that structural growth is irreversible (*C*_growth_≥0). A breakdown of soma, e.g. muscle tissue during starvation as seen in nature, is thus restricted to the part included in the reserves.

To meet the requirements of different tissues, nutrients have to be converted, and conversion of metabolites comes with a cost. When storing energy, processing of nutrients into storage molecules is based on a conversion efficiency *k*_conversion_reserves_ [dimensionless]. The model assumes this conversion to be biochemical processes that requires oxygen and therefore will contribute to overall metabolic rate:(17)
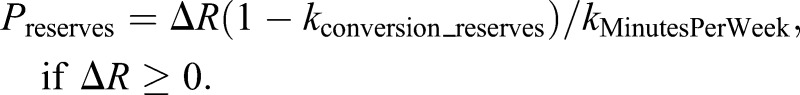
If energetic expenses exceed the energy available from digestion, reserves have to be drained. Then a conversion cost has to be paid for making those reserves accessible:(18)
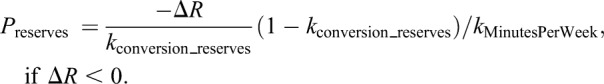
In the case of growth, metabolites are drawn from reserves and converted into building blocks. The cost *P*_growth_ of conversion into growth is also calculated using a conversion efficiency parameter *k*_conversion_growth_ [dimensionless]:(19)
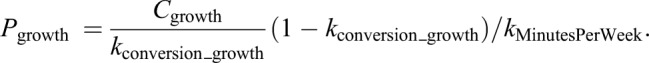


### Aerobic scope

The maximum rate of oxygen uptake has to accommodate all oxygen-dependent processes such as digestion, locomotion, foraging, conversion of energy and other metabolic activities (Fry, 1971). We refer to the unused surplus as the free aerobic scope (Holt and Jørgensen, 2015).

We calculate potential oxygen uptake *A*_standard_ [J min^−1^] following Claireaux et al. (2000) as an allometric function with exponent *b*<1. Because it is unrealistic that variations in reserve size affect an individual's capacity for oxygen uptake, we base calculations of aerobic scope on the structural body mass only:(20)
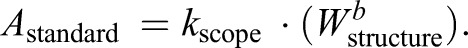
Here, *k*_scope_ has unit [J min^−1^ g^−*b*^].

A key assumption of our model is that the THF increases aerobic scope through increasing capacity for oxygen uptake, thus permitting higher levels of metabolic processes, but at a cost on SMR (Eqn 8):(21)
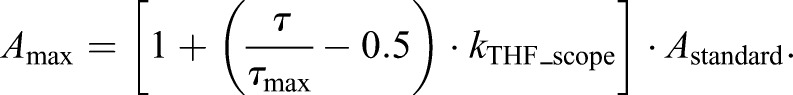
Here, *k*_THF_scope_ [dimensionless] sets the strength of the effect of THF on increased scope.

### Food availability

Across model runs we vary food availability, implemented as the factor *E* [dimensionless]. When food availability is good (high *E*), less foraging activity is required to obtain the given amount of resources (Eqn 11). Contrary, when *E* is low, the individual needs more time to gather the amount of food it aims for. Consequently, *E*, through *B*_foraging_, determines the exposure to predators in Eqn 23, and the energetic cost of foraging in Eqn 12. In this version of the model, there is no stochasticity influencing foraging success.

### Mortality rates

In this model, mortality is decompounded into discrete risk factors (Eqn 4) that through separate trade-offs contribute to an individual's risk of being depredated or otherwise die (extended from Holt and Jørgensen, 2014). All mortality rates (with capitalized *M*) are in unit year^−1^, while the units of the various constants introduced (with small *m*) are given in Table S1. The first is a constant component *m*_fixed_ represents death due to causes that are independent of the individual's state or behaviour, e.g. some types of disease. Second is size-dependent mortality, with reduced risk of mortality with larger body size, as is both observed (Gislason et al., 2010; Peterson and Wroblewski, 1984) and resulting from the size-structure of marine food webs and scaling relationships (Brown et al., 2004). We model this as an allometric relationship with a negative exponent:(22)
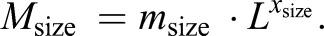
The next mortality component reflects the well-known trade-off between risk of predation and foraging intensity (e.g. Lima, 1998). The model assumes that individuals expose themselves to predation risk while foraging, and that this risk accelerates with increasing foraging because the safest habitats and time periods are assumed exploited first:(23)
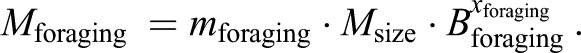
For this and the risk components below, it is assumed that predation is the ultimate cause for death and therefore that the risk declines with size in the same way as the size-dependent predation mortality.

The final two components relate to oxygen use and aerobic scope, i.e. the difference between maximum oxygen uptake and actual rate of oxygen use. Fleeing from predators demands burst swimming, which is achieved anaerobically by white muscle (Johnston, 1981; Rome et al., 1988; Weber et al., 2016). Recovery is aerobic and faster if there is free aerobic scope to provide abundant oxygen (Killen et al., 2014; Marras et al., 2010), thus preparing the individual for a repeated attack or the next encounter. We model this based on the ratio between used and available oxygen, raised to a power to describe how predation risk increases rapidly as maximum oxygen uptake is approached or even temporarily exceeded:(24)
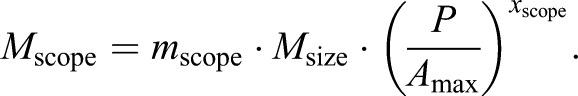
The model finally assumes that it is particularly risky for an individual to expose itself (high *M*_foraging_) when oxygen use is high (high *M*_scope_) because attacks would be frequent and recovery at the same time slow:(25)
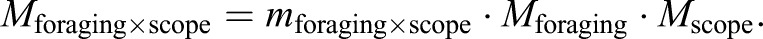
The mortality rates [year^−1^] stemming from each risk factor are then summed (Eqn 4) and survival per time step [week] given as *S*=e^−*M*/52^.

### Implementation

The model follows juvenile fish as they grow from 10 cm to 30 cm body length. Optimal solution is found for each combination of the individual states length (21 steps) and reserves (10 steps). Hormone levels are discretized into 160 each. Time step is 1 week, and we allow sufficient time horizon for all fish to reach maximum size, which normally takes less than 200 weeks for the slowest ones.

### Parameterization

Parameters used in the model were chosen from different fish species to create a generalized, juvenile fish. Many of the studies used were performed on Atlantic cod, which makes cod the fish most similar to the model fish.

For orexin A no studies on hormone concentrations in fish are known. In this case measurements on mammals were used.

The water temperature was set constant at 5°C and water was assumed saturated with oxygen.

Energy density for reserves was chosen to be 5000 J/g. This is based on a calculation of mean protein and fat contents in storage tissues. A fish of 750 g served as a template. Energy density was based on the weight of liver and white muscle tissue and their proportional content of fat and proteins. For proteins, cellular water was taken into account.

Since growth requires development of more specialized tissue than storing molecules in reserves, the conversion efficiency for growth was set lower than for reserves.

Fulton's condition factors for fish with full reserves (*k*_Fultons_max_) and depleted reserves were chosen following a study on cod (Lambert and Dutil, 1997b).

Variables used in calculations of SMR (*k*_SMR_, *α*) were based on Clarke and Johnston (1999), Mozsár et al. (2015) and Pangle and Sutton (2005), describing the resting metabolic rate of a general teleost fish. In line with earlier models built on a similar bioenergetics template (e.g. Jørgensen and Fiksen, 2010), we used a scaling exponent *a*=0.7, which is within the range of intraspecific scaling exponents among teleosts (Killen et al., 2007). Also, studies show that there is great variation in scaling exponents in animals and the value chosen here was in the range of this variation (Holdway and Beamish, 1984; Kjesbu et al., 1991; Lambert and Dutil, 1997a). Units were converted to fit the model.

The coefficient *k*_scope_ used in calculations was derived from a study on cod (Claireaux et al., 2000). The scaling exponent for aerobic scope (*b*) was chosen in accordance with SMR scaling (Holt and Jørgensen, 2014).

### Hormone concentrations

Concentrations of IGF-1 were given in ng/ml blood plasma and the model allowed a range from 0–200. In experiments with tilapia concentrations of 70–120 ng/ml plasma were measured (Uchida et al., 2003). A study on Arctic char revealed concentrations up to approximately 250 ng/ml plasma (Cameron et al., 2007).

Orexin A has been detected in concentrations up to roughly 350 pg/ml porcine blood plasma (Kaminski et al., 2013), which is a range assumed to be normal for adult men and women (Oka et al., 2004). The range is higher for children, where measurements up to roughly 1300 pg/ml have been observed (Tomasik et al., 2004). The model allowed orexin A up to 2000 pg/ml blood plasma. Its existence and function in fish has mainly been documented in goldfish (Abbott and Volkoff, 2011; Hoskins et al., 2008; Volkoff et al., 1999) and zebrafish (Matsuda et al., 2012).

Concentrations of T_3_ were given in ng/ml of blood plasma and range of 0–5. The range was chosen according to measurements on teleosts, e.g. 1-year-old rainbow trout (*Oncorhynchus mykiss*) (Eales, 1988), *Anabas testudineus* (Varghese and Oommen, 1999; Varghese et al., 2001) and chum salmon (*Oncorhynchus keta*) (Tagawa et al., 1994), which revealed concentrations up to roughly 4.5 ng/ml plasma for normal individuals.

## Supplementary Material

Supplementary information
