# Supplementary material for: Hormones as adaptive control systems in juvenile fish
Source: Biol Open. 2020 Feb 17;9(2):bio046144. doi: 10.1242/bio.046144 (PMC7044463; doi:10.1242/bio.046144)
Supplement: Supplementary information [file biolopen-9-046144-s1.pdf]

Table S1. Parameters used in the growth model of a generalized fish using hormonal strategies to adapt to environmental challenges.

**Parameters**				
**Name**	**Value**	**Unit**	**Definition**	**Literature**
a	0.7	-	Exponent for standard metabolic rate	(Clarke and Johnston, 1999)
b	0.7	-	Exponent for calculation of maximum aerobic scope	-
d_{reserves}	5 000	J g^{-1}	Energy density of reserves	-
$d_{\text{structure}}$	4 000	J g^{-1}	Energy density of soma	-
$k_{\text{conversion\_growth}}$	0.75	-	Efficiency of converting metabolites from reserves to soma	-
$k_{\text{conversion\_reserves}}$	0.85	-	Efficiency of converting metabolites between blood and reserves	-
k_{foraging}	0.2	-	Scaling factor for energetic cost of foraging	-
k_{growth}	0.28	-	Upper limit for proportional increase in structural body mass	-
$k_{\text{Fultons\_max}}$	1.2·10^{-8}	0.01 g cm^{-3}	Fulton’s condition factor for fish with full reserves	(Lambert and Dutil, 1997b)
$k_{\text{ Fultons\_min}}$	0.85·10^{-8}	0.01 g cm^{-3}	Fulton’s condition factor for lean fish	(Lambert and Dutil, 1997b)
$k_{\text{MinutesPerWeek}}$	10080	-	Number of minutes in one time step	-
k_{OXF}	5	-	Scaling factor for effect of OXF on intake (including urinary and fecal energy loss)	-
k_{scope}	2.58·10^{-5}	J min^{-1} g^{-b}	Coefficient for calculation of maximum aerobic scope	(Claireaux et al., 2000)
k_{SDA}	0.15	-	Coefficient for calculation of SDA	-
k_{SMR}	89596.7	J min^{-1} g^{-a}	Scaling factor for standard metabolic rate	(Clarke and Johnston, 1999)
$k_{\text{THF\_scope}}$	0.24	-	Scaling factor determining the strength of THF on AMR	-
$k_{\text{THF\_SMR}}$	0.23	-	Scaling factor determining the strength of THF on SMR	-
m_{fixed}	0.01	year^{-1}	Background mortality rate (constant)	-
m_{foraging}	0.08	-	Coefficient for calculation of foraging-related mortality rate	-

Biology Open • Supplementary information

$m_{\mathrm{foraging}\times\mathrm{scope}}$	0.9	year	Coefficient for calculation of active-while-vulnerable mortality rate	-
m_{scope}	0.8	-	Coefficient for calculation of scope-related mortality rate	-
m_{size}	0.038	year^{-1} $\mathrm{cm}^{-x_{\mathrm{size}}}$	Coefficient for calculation of size-dependent mortality rate	-
x_{foraging}	2	-	Exponent for calculation of foraging-related mortality rate	-
x_{scope}	3	-	Exponent for calculation of scope-related mortality rate	-
x_{size}	-0.75	-	Exponent for calculation of size-dependent mortality rate	-
α_{max}	1500	pg ml^{-1}	Maximum value of OXF	-
γ_{max}	200	ng ml^{-1}	Maximum value of GHF	-
τ_{max}	5	ng ml^{-1}	Maximum value of THF	-

Biology Open • Supplementary information

Table S2. Variables used in a state-dependent fish growth model using optimized hormonal strategies.

**Variables**		
**Name**	**Unit**	**Definition**
A_{max}	J min^{-1}	Maximum aerobic scope under influence of THF
A_{standard}	J min^{-1}	Maximum aerobic scope (AMR)
B_{foraging}	-	Foraging behaviour
C	J min^{-1}	Energetic costs of building new tissue (soma and reserves)
C_{growth}	J	Energy incorporated in new structural tissue
E	-	Food abundance in environment
I	J min^{-1}	Intake (corresponds to metabolizable energy)
L	cm	Body length
M	year^{-1}	Total mortality rate
M_{foraging}	year^{-1}	Foraging-related mortality rate
$M_{\mathrm{foraging\times scope}}$	year^{-1}	Active-while-vulnerable mortality rate
M_{scope}	year^{-1}	Scope-related mortality rate
M_{size}	year^{-1}	Size-dependent mortality rate
P	J min^{-1}	Metabolic processes
P_{foraging}	J min^{-1}	Swimming cost of foraging behaviour
P_{growth}	J min^{-1}	Cost of converting metabolites from reserves into new structural tissue
P_{reserves}	J min^{-1}	Cost of converting metabolites from bloodstream into fat and proteins for storage
P_{SDA}	J min^{-1}	Cost of digestion and energy uptake into bloodstream
P_{SMR}	J min^{-1}	Standard metabolic rate (SMR) under influence of THF
P_{standard}	J min^{-1}	Standard metabolic rate (SMR)
$P_{\mathrm{structure}}$	J min^{-1}	Standard metabolic rate based on structural weight
R	J	Energy reserves
R_{max}	J	Maximum reserves depending on body size
ΔR	J	Energy incorporated in reserves (when negative, reserves are drained)
S	year^{-1}	Survival probability
W	g	Body mass (structural and reserves)
$W_{\mathrm{structure}}$	g	Structural body mass
$\Delta W_{\mathrm{structure}}$	g week^{-1}	Growth
α	pg ml^{-1}	Level of OXF
γ	ng ml^{-1}	Level of GHF
τ	ng ml^{-1}	Level of THF

Biology Open • Supplementary information

Table S3

Click here to download Date Code S1

Table S4

Click here to download Date Code S2
